# Controlled Amphiphilicity and Thermo-Responsiveness of Functional Copolymers Based on Oligo(Ethylene Glycol) Methyl Ether Methacrylates

**DOI:** 10.3390/polym16111456

**Published:** 2024-05-22

**Authors:** Aggeliki Christopoulou, Charalampos Kazamiakis, Zacharoula Iatridi, Georgios Bokias

**Affiliations:** 1 Department of Chemistry, University of Patras, GR-26504 Patras, Greece; up1064175@ac.upatras.gr (A.C.); up1073632@ac.upatras.gr (C.K.); bokias@upatras.gr (G.B.); 2 Department of Materials Science, University of Patras, GR-26504 Patras, Greece; 3 Foundation for Research and Technology Hellas (FORTH), Institute of Chemical Engineering and High Temperature Chemical Processes, GR-26504 Patras, Greece

**Keywords:** oligo(ethylene glycol) methyl ether methacrylate, glycidyl methacrylate, N,N-dimethylacrylamide, thermo-responsiveness, self-assembly, functional copolymers

## Abstract

In this work, comb homopolymers as well as comb-type copolymers of thermo-responsive oligo(ethylene glycol methyl ether methacrylate)s, OEGMAs, with various chain lengths (DEGMA, PEGMA_500_, and PEGMA_950_ containing 2, 9, or 19 repeating ethylene glycol units, respectively) were synthesized through free radical (co)polymerization. For the copolymers, either the functional hydrophobic glycidyl methacrylate (GMA) or the inert hydrophilic N,N-dimethylacrylamide (DMAM) were selected as comonomers. The self-assembly and thermo-responsive behavior of the products was investigated through Nile Red fluorescence probing, turbidimetry, and dynamic light scattering (DLS). Interestingly, it was found that all OEGMA-based homopolymers exhibit a tendency to self-organize in aqueous media, in addition to thermo-responsiveness. The critical aggregation concentration (CAC) increases with the number of repeating ethylene oxide units in the OEGMA macromonomers (CAC was found to be 0.003, 0.01, and 0.03% *w*/*v* for the homopolymers PDEGMA, PPEGMA_500_, and PPEGMA_950_, respectively). Moreover, the CAC of the copolymers in aqueous media is highly affected by the incorporation of hydrophobic GMA or hydrophilic DMAM units, leading to lower or higher values, respectively. Thus, the CAC decreases down to 0.003% *w*/*v* for the GMA-richest copolymer of PEGMA_950_, whereas CAC increases up to 0.01% *w*/*v* for the DMAM-richest copolymer of DEGMA. Turbidimetry and DLS studies proved that the thermo-sensitivity of the polymers is governed by several parameters such as the number of repeating ethylene glycol groups in the side chains of the OEGMAs, the molar percentage of the hydrophobic or hydrophilic comonomers, along with the addition of salts in the aqueous polymer solutions. Thus, the cloud point of the homopolymer PDEGMA was found at 23 °C and it increases to 33.5 °C for the DMAM-richest copolymer of DEGMA. Lastly, the formation of a hydrogel upon heating aqueous mixtures of the GMA-comprising copolymers with silica nanoparticles overnight is strong evidence of the functional character of these polymers.

## 1. Introduction

Stimuli-responsive polymers, often called “smart” polymers, are materials that undergo changes to their physicochemical characteristics and/or structural conformations upon exposure to external stimuli [1,2]. Such stimuli may be chemical like pH [3], ionic strength [4], and chemicals/solvents [5], physical such as temperature [6,7], light [8], and mechanical stress [9], and biological like enzymes and oxidation [10,11]. Smart polymers can have numerous applications like drug delivery, sensing, biomedical applications, etc. [2]. Thermo-responsive polymers are materials, the physical properties of which are affected by changes in temperature [6]. One of the most popular and studied thermo-responsive polymers is poly(N-isopropylacrylamide) (PNIPAM) [12] which belongs to the class of polyacrylamides. The thermo-responsiveness of PNIPAM in aqueous solutions is attributed to its characteristic lower critical solution temperature (LCST) around 32 °C. Below its LCST, PNIPAM is soluble in water, while above its LCST, it becomes hydrophobic and phase separates. Other thermo-responsive polymers that have been extensively studied are polyoxazolines [13], while special attention has been devoted over the last two decades to another class of polymers with thermo-sensitive behavior, namely the homopolymers or copolymers of oligo(ethylene glycol) acrylates (OEGAs) or oligo(ethylene glycol) methyl ether methacrylates (OEGMAs) [14,15,16,17,18]. 

Usually, polymers based on OEGMAs are applied as substitutes for linear polyethylene glycol (PEG). Such (co)polymers are preferred to PNIPAM and they can find use in bioapplications [19,20,21] since they are non-toxic, biocompatible, and non-immunogenic [22,23]. Their LCST depends strongly on the length of the ethylene glycol (EG) brushes [24,25,26]. For example, it has been found that when comparing aqueous solutions of POEGMAs with two, three, five, and nine repeating ethylene glycol (EG) groups, the longer the OEGMA brush, the more hydrophilic the polymers are and consequently the higher the cloud point temperature (Tcp) observed [27]. 

The LCST of POEGMAs can also be precisely controlled to the application requirements by copolymerizing OEGMAs with hydrophilic or hydrophobic monomers. Copolymerization with hydrophilic monomers leads to final copolymers with higher LCST values, while copolymerization with hydrophobic monomers shifts the LCST of POEGMAs to lower values because of the enhanced hydrophobicity of the system in aqueous solutions. Indeed, it has been shown that the copolymerization of various alkyl methacrylates (RMA, R = methyl, ethyl, n-butyl, n-hexyl, and n-dodecyl) with OEGMA (with eight repeating EG units) leads to a series of copolymers with lower cloud point temperatures (Tcp) compared to the POEGMA homopolymer [28]. In another work, the Tcp of statistical copolymers of OEGMA (with eight repeating EG units in its side chains) with the hydrophobic styrene could be easily adjusted to lower values by simply changing the initial styrene monomer feed in the polymerization mixture [29]. On the other hand, it has been reported that for random copolymers of di(ethylene glycol)ethyl ether acrylate (DEGA) with the hydrophilic N,N-dimethylacrylamide (DMAM), the cloud point temperature (Tcp) of the polymers exhibited an increase in the range ~20 to ~90 °C upon increasing the molar percentage of DMAM from 20 to 80% [30]. Moreover, block-type (synthesized via atom transfer radical polymerization, ATRP) [31] or random/gradient (synthesized via reversible addition–fragmentation chain transfer polymerization, RAFT) [32,33,34] copolymers of di(ethylene glycol) methyl ether methacrylate (DEGMA) and other OEGMA comonomers with different lengths in their side chains have been investigated. The Tcp values of these copolymers are between the Tcps of the respective PDEGMA and POEGMA homopolymers, showing a linear increase with the molar fraction of OEGMA units.

Our group has long experience in the design and study of thermo-responsive NIPAM-based copolymers [35,36,37]. Moreover, we have recently reported the synthesis of random copolymers of an OEGMA macromonomer (PEGMA_950_, with 19 repeating EG groups in its side chains) with the hydrophobic monomer glycidyl methacrylate (GMA) and studied their potential use as healing agents of waterborne polyurethanes [38]. Aimed at the development of functional thermo-responsive polymeric materials, the synthesis and behavior in aqueous solution of a series of OEGMA-based homopolymers and amphiphilic copolymers with the hydrophobic GMA monomer is presented in this work. Three OEGMA macromonomers with various chain lengths: 2 (DEGMA, short), 9 (PEGMA_500_, medium), and 19 (PEGMA_950_, long) repeating EG groups in their side chains were polymerized through an easy, free radical polymerization method. For reasons of comparison, copolymers of DEGMA with the hydrophilic nonionic monomer N,N-dimethylacrylamide (DMAM) were also prepared. The self-organization and thermo-sensitivity of the (co)polymers are explored as a function of the lengths of the OEGMA macromonomers, the nature of the comonomer (hydrophilic DMAM or hydrophobic GMA), as well as the ionic strength of the aqueous solution using kosmotropic or chaotropic salts. 

It should be mentioned that, though several structural designs have been elaborated and the respective studies of the thermo-responsive properties of such OEGMA-based copolymers are numerous, studies on self-organization behavior (with a focus on critical aggregation concentration) are very rare, to our knowledge. In the present work, the combined investigation of both properties, self-organization and thermo-sensitivity, as a function of the chemical structure of the copolymers is attempted, aiming at a more complete overall picture. In addition, the functional character of GMA-containing copolymers is revealed through rheology in aqueous copolymer/silica nanoparticle mixtures after heating at 80 °C to form polymer/silica nanoparticle hybrids. This, in fact, offers a facile approach toward the formation of OEGMA-decorated silica nanoparticle dispersions, hydrogels, and coatings. Such materials can find applications in several fields, for example, as emulsifiers for the formation of Pickering emulsions [39] or as thermo-responsive hybrid coatings with protein resistance [40]. 

## 2. Materials and Methods

### 2.1. Materials

The monomers di(ethylene glycol) methyl ether methacrylate (DEGMA, >97%), poly(ethylene glycol) methyl ether methacrylate (PEGMA_500_, approximately 9 EG units in each side chain, Mn = 500 Da), glycidyl methacrylate (GMA, >95%), and N,N-dimethylacrylamide (DMAM, 99%) were used as received from TCI chemicals (Zwijndrecht, Belgium). The solvents tetrahydrofuran (THF, anhydrous, p.a, ≥99.9%) hexane (p.a, ≥99.5%) and diethyl ether (p.a, ≥99.5%) were supplied by Carlo Erba (Milan, Italy), while the solvent deuterated chloroform (CDCl_3_, 99.8%D) was obtained from Eurisotop (Saint-Aubin, France). The initiator azobisisobutyronitrile (AIBN) and Nile Red (technical grade) were obtained from Aldrich (Steinheim, Germany). Na_2_SO_4_ and KSCN were supplied by Fisher Chemical (Waltham, MA, USA) and Aldrich (Steinheim, Germany), respectively. Fumed silica nanoparticles (0.2–0.3 μm) were supplied by Aldrich (Steinheim, Germany). Ultrapure water was prepared using an Arium mini water purification system (Sartorius, Göttingen, Germany). 

### 2.2. Synthesis of POEGMA Homopolymers

Three homopolymers based on three OEGMA macromonomers with different molecular weights (Mn) were synthesized through conventional free radical polymerization: the homopolymers originating from the OEGMAs with Mn = 188 Da, Mn = 500 Da, and Mn = 950 Da are denoted herein as PDEGMA, PPEGMA_500_, and PPEGMA_950_, respectively. In each synthesis, the OEGMA monomer was dissolved in THF (total monomer’s concentration in THF: 20% *w*/*v*). The solution was degassed with nitrogen and the initiator AIBN (1 mol% over the total monomer concentration) was added. The polymerization reaction was carried out for 24 h in an oil bath at 70 °C and in a nitrogen atmosphere. The reaction mixture was then precipitated in excess volume of hexane or diethyl ether. The precipitated homopolymer was subsequently washed with hexane and lastly dried in a vacuum oven at 40 °C.

### 2.3. Synthesis of P(OEGMA-co-GMA) Copolymers 

The copolymers poly(oligo(ethylene glycol) methyl ether methacrylate-co-glycidyl methacrylate) were synthesized through free radical cοpolymerization, using AIBN as an initiator. The copolymers of GMA with PEGMA_500_ or PEGMA_950_ are denoted as P(PEGMA_x_-co-GMAy), where x is the Mn of OEGMA units. The copolymers of DEGMA with GMA are denoted as P(DEGMA-co-GMAy). In all cases, y is the mol fraction of GMA units, in the copolymers, as determined via ^1^H-NMR characterization in CDCl_3_. The synthesis of all copolymers was performed following the protocol described elsewhere for the two P(PEGMA_950_-co-GMAy) copolymers with y = 54 and 74% moles [38]. Briefly, the desired quantity of the two monomers was dissolved in THF (total monomer concentration in THF: 10% *w*/*v*), and the solution was degassed by nitrogen, followed by the addition of the initiator AIBN (1 mol% over the total monomer concentration). The reaction was left to proceed overnight under vigorous stirring in nitrogen atmosphere in an oil bath set at 70 °C. After cooling down to room temperature, the copolymers were recovered via precipitation and successive washing with hexane or diethyl ether, filtered, and dried in a vacuum oven at 40 °C for 24 h.

### 2.4. Synthesis of P(DEGMA-co-DMAM) Copolymers

Two poly(di(ethylene glycol) methyl ether methacrylate-co-N,N-dimethylacrylamide) copolymers were similarly synthesized, as described above. The copolymers are denoted as P(DEGMA-co-DMAMy), where y is the mol fraction of DMAM units, in the copolymers, as determined via ^1^H-NMR characterization in CDCl_3_. The copolymers were synthesized in THF (total monomer concentration in THF: 10% *w*/*v*), adding AIBN (1 mol% over the total monomer concentration) at 70 °C under nitrogen. After cooling down, the P(DEGMA-co-DMAMy) copolymers were recovered via precipitation in hexane, washed with excess volumes of hexane, filtered, and finally dried in a vacuum oven at 40 °C for 24 h.

### 2.5. Characterization of Copolymers

Characterization of the polymers with Proton Nuclear Magnetic Resonance (^1^H-NMR) in CDCl_3_ was achieved by means of a Bruker AVANCE DPX 400 spectrometer (Billerica, MA, USA) (400 MHz, 25 °C). Attenuated total reflection–Fourier transform infrared (ATR-FTIR) spectra of the polymers were recorded on a Bruker Platinum ATR-FTIR spectrometer (Billerica, MA, USA). 

### 2.6. Determination of Critical Aggregation Concentration (CAC)

Nile Red fluorescence probing was used to determine the critical aggregation concentration (CAC) of the polymers. For this, a Perkin Elmer LS50B luminescence spectrometer was used (Waltham, MA, USA). Aqueous polymer solutions with concentrations varying from 10^−4^ to 1% *w*/*v* were prepared. 5 μL of a stock THF solution, containing 10^−3^ M Nile Red, was added to 3 mL of each aqueous polymer solution. From the fluorescence spectra, the maximum intensity of the emission peak of Nile Red in the region of 600–650 nm, after excitation at 550 nm, was used to detect the formation of the hydrophobic polymer microdomains. The excitation and emission slits were fixed at 10 nm.

### 2.7. Thermo-Responsive Behavior

For cloud point temperature (Tcp) determination, the optical density at 500 nm was examined using a HITACHI U-1800 spectrophotometer (Schaumburg, IL, USA). For this study, 0.2% *w*/*v* aqueous solutions of the polymers were prepared, in pure water or in the presence of Na_2_SO_4_ or KSCN salts (0.25 M or 0.5 M). Tcp was defined as the temperature of the onset of optical density, reflecting the onset of turbidity. The effect of temperature on the hydrodynamic diameter of the polymer assemblies in water was studied using a ZetaSizer Nano series Nano-ZS (Malvern Instruments Ltd., Worcestershire, UK) equipped with a He-Ne Laser beam at a wavelength of 633 nm and a fixed backscattering angle of 173°. 

### 2.8. Rheology Study 

Aqueous solutions of P(PEGMA_950_-co-GMA54) copolymer were mixed with aqueous dispersions of silica nanoparticles via magnetic stirring. The final polymer and silica concentrations were set at 5%wt. and 3.3%wt., respectively. The polymer/silica nanoparticle suspensions were either left under RT conditions or heated overnight at 80 °C using an oven. Thereafter, all sample suspensions were studied with an AR-2000ex stress-controlled rheometer (TA Instruments, New Castle, DE, USA) equipped with a Peltier system for controlling the temperature. A cone–plate geometry (diameter 20 mm, angle 3°, and truncation 111 μm) was used. The studies were performed at 25 °C. 

## 3. Results and Discussion

### 3.1. Synthesis and Characterization of Copolymers

**Figure 1:**
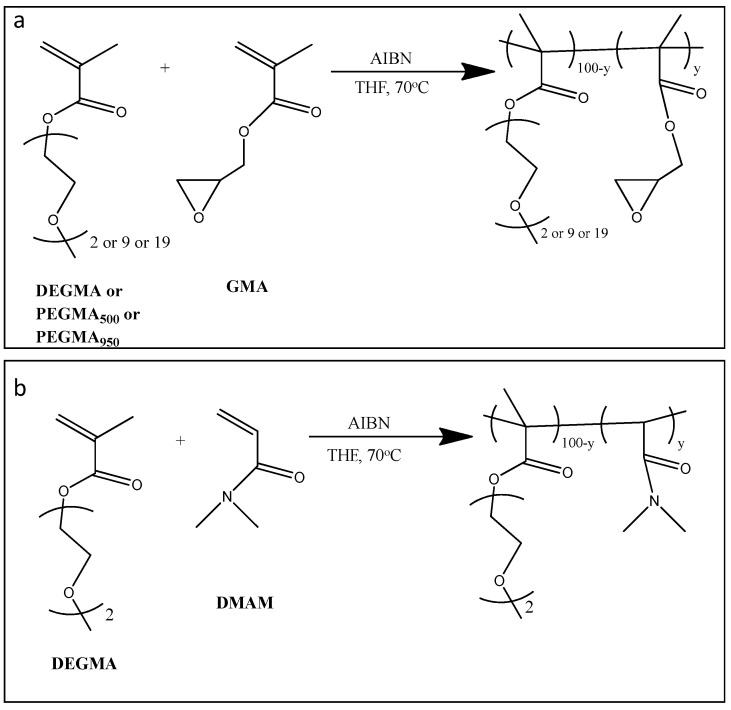
Synthesis route of (**a**) copolymers of GMA with DEGMA, PEGMA_500_, or PEGMA_950_ and (**b**) copolymers of DEGMA with DMAM.

The free radical polymerization method was applied for the synthesis of homopolymers and copolymers based on oligo(ethylene glycol) methyl ether methacrylates (OEGMAs), using AIBN as an initiator. In Figure 1, the synthesis routes of the P(OEGMA-co-GMAy) and P(DEGMA-co-DMAMy) copolymers are presented. 

PPEGMA_500_ and P(PEGMA_500_-co-GMAy) polymers are transparent, viscous liquids, while PDEGMA and P(DEGMA-co-DMAMy) are soft, transparent materials. All other polymers, (P(DEGMA-co-GMA12), PPEGMA_950_, and P(PEGMA_950_-co-GMAy)), are opaque, waxy, solid materials. 

**Figure 2:**
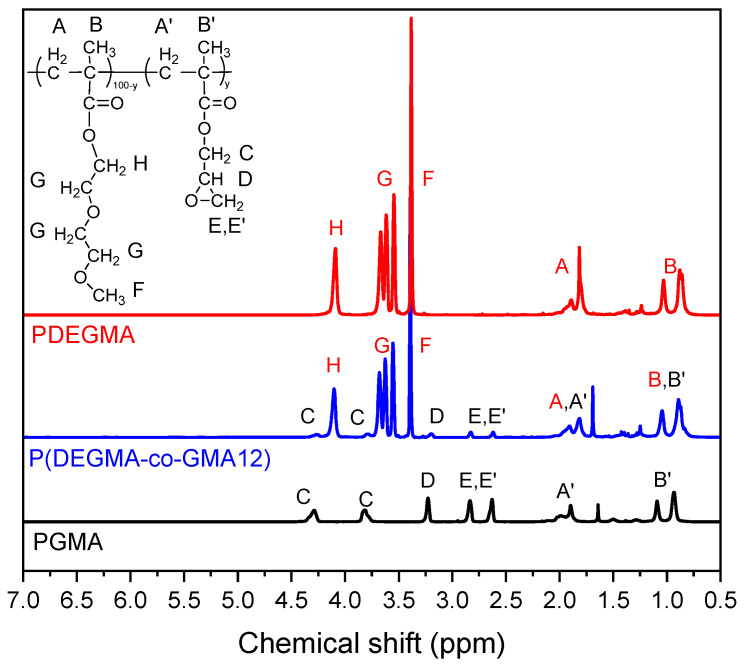
^1^H-NMR spectra of PDEGMA, PGMA, and P(DEGMA-co-GMA12).

All OEGMA-based copolymers were characterized via proton nuclear magnetic resonance (^1^H-NMR) and attenuated total reflection–Fourier transform infrared spectroscopy (ATR-FTIR). The synthesis and characterization of P(PEGMA_950_-co-GMAy) copolymers have already been reported [38]. The ^1^H-NMR spectra of the P(PEGMA_500_-co-GMAy) copolymers are presented in Figure S1 along with the respective spectra of the PPEGMA_500_ and PGMA homopolymers. In Figure 2, the ^1^H-NMR spectrum of the P(DEGMA-co-GMA12) copolymer in CDCl_3_ is compared with the spectra of the two homopolymers, PDEGMA and PGMA. The broad signals “A, A′” (at ~2 ppm) and “B, Β′” (~1 ppm) are related to the methylene (–CH_2_–) and methyl protons (–CH_3_), respectively, of the main chain of both DEGMA and GMA units. As far as the side chains of DEGMA are concerned, a strong signal of the O-CH_3_ “F” protons was observed at 3.4 ppm, while the “H” and “G” protons were detected at 4.2 ppm and ~3.6–3.9 ppm, respectively. In the spectrum of PGMA, the protons of the –CH_2_– group of the epoxide ring of GMA “E, E΄” were detected at 2.7 and 2.9 ppm, while the proton of the –CH– group “D” was identified at 3.26 ppm. Moreover, the signals at 3.8 and 4.3 ppm correspond to the methylene protons –O–CH_2_– “C” adjacent to the epoxy groups of GMA. The presence of all characteristic peaks from both DEGMA and GMA in the spectrum of P(DEGMA-co-GMA12) proves its successful copolymerization. From this spectrum, the monomer molar ratio was determined by comparing the signals at 3.4 ppm (F protons from the DEGMA unit) and 3.26 ppm (D proton from the GMA unit). The resulting composition of the copolymer is in rather good agreement with the feed composition (Table 1). 

**Figure 3:**
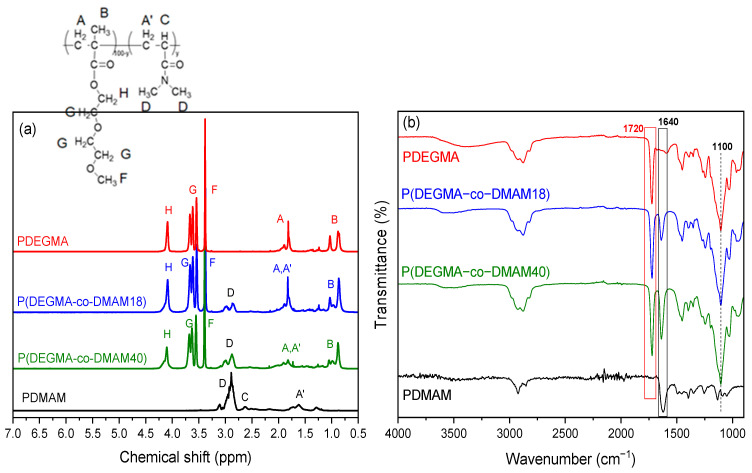
(**a**) ^1^H-NMR and (**b**) ATR-FTIR spectra of PDEGMA, PDMAM, P(DEGMA-co-DMAM18), and P(DEGMA-co-DMAM40).

The ^1^H-NMR of P(DEGMA-co-DMAMy) copolymers are presented in Figure 3a. Together, we present the spectra of PDEGMA and PDMAM homopolymers for comparison. As can be seen, in the spectra of both P(DEGMA-co-DMAMy) copolymers, all characteristic peaks of DEGMA were identified. Moreover, the representative signal “D” (2.8–3.2 ppm), attributed to the six protons of methyl (–CH_3_) groups connected with the nitrogen atom in the DMAM units, are also seen in the spectra of the copolymers, indicating the effective copolymerization of DEGMA with DMAM. The integrals of the signals at 3.4 ppm (F protons of DEGMA) and 2.8–3.2 ppm (D protons of DMAM units) were used to calculate the molar ratio of DEGMA and DMAM in the P(DEGMA–co–DMAMy) copolymers. The results are summarized in Table 1. 

The ATR-FTIR characterization of the P(DEGMA-co-DMAMy) copolymers is presented in Figure 3b. The characteristic band of the polymers that appears from 2800–3000 cm^−1^ corresponds to the stretching vibrations of C-H groups. The signal of this area is somewhat more intense in the case of the P(DEGMA-co-DMAMy) copolymers, suggesting successful copolymerization. The signal in the region 3200–3700 cm^−1^ is related to the presence of moisture which is due to the hydrophilic nature of the polymers. The characteristic peaks at 1720 cm^−1^, 1250 cm^−1^, and 1100 cm^−1^, corresponding to the stretching vibrations of the ester groups (C=O) and the stretching vibration of the ether bonds (C-O-C) from DEGMA, are present in the copolymers’ spectra. Moreover, the characteristic signal at the peak at 1640 cm^−1^, owing to the amide groups of DMAM, was also detected in the spectra of P(DEGMA-co-DMAMy) polymers, indicating that the copolymerization of DEGMA with DMAM was achieved. The relative intensity of these peaks agrees with the increase in the DMAM content of the copolymers.

### 3.2. Self-Assembly of Polymers in Aqueous Solution

**Figure 4:**
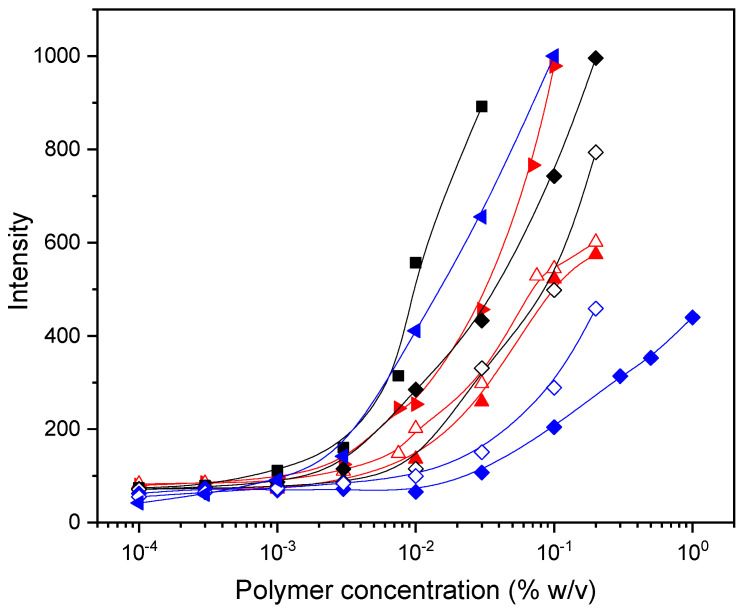
Intensity at 625 nm as a function of the concentration of PDEGMA (■), P(DEGMA-co-DMAM18) (♦), P(DEGMA-co-DMAM40) (◊), PPEGMA_500_ (▲), P(PEGMA_500_-co-GMA26) (∆), P(PEGMA_500_-co-GMA50) (►), PPEGMA_950_ (♦), P(PEGMA_950_-co-GMA54) (◊), and P(PEGMA_950_-co-GMA74) (◄).

One of the most characteristic behaviors of water-soluble amphiphilic copolymers comprising hydrophilic and hydrophobic segments is their tendency to self-associate in water into various assemblies including micelles, flower-like micelles, vesicles, bilayers, large compound micelles, etc., via the association of the hydrophobic segments and exposing to the aqueous environment the hydrophilic segments [41,42]. Further increasing the content of hydrophobic segments leads to amphiphilic polymers with decreased CAC values [43]. In our (co)polymers, the hydrophilic segments are the ethylene glycol side chains, while the apolar carbon–carbon backbone (together, eventually, with GMA units) comprises the hydrophobic segments. It is thus expected that the incorporation of the hydrophobic GMA units along the OEGMA side chains will affect the self-assembly properties of the copolymers in aqueous media. Fluorescence probing using Nile Red as a fluorescence probe was used to study the self-association of the synthesized polymers in aqueous solution. Nile Red is a poorly water-soluble probe molecule that is almost non-fluorescent in water. In addition, it undergoes a change in fluorescence intensity or emission wavelength (it exhibits an intense emission peak in the region of 600–650 nm) upon solubilization in the hydrophobic cores (less polar environments) of formed micelles/aggregates. In Figure 4, the maximum emission intensities of Nile Red at 600–650 nm are plotted versus the polymer concentrations. As seen, for all studied polymers, the maximum intensity of Nile Red is low at low polymer concentrations since the probe detects purely hydrophilic environments. Above a certain polymer concentration (called the critical aggregation concentration, CAC), the intensity of Nile Red increases sharply. This increase is evidence of the formation of self-associated structures by the polymer chains, which probably consist of hydrophobic cores surrounded by hydrophilic units, stabilizing the system in the aqueous media. Nile Red is then solubilized in these hydrophobic microenvironments, leading to enhanced fluorescence. 

**Figure 5:**
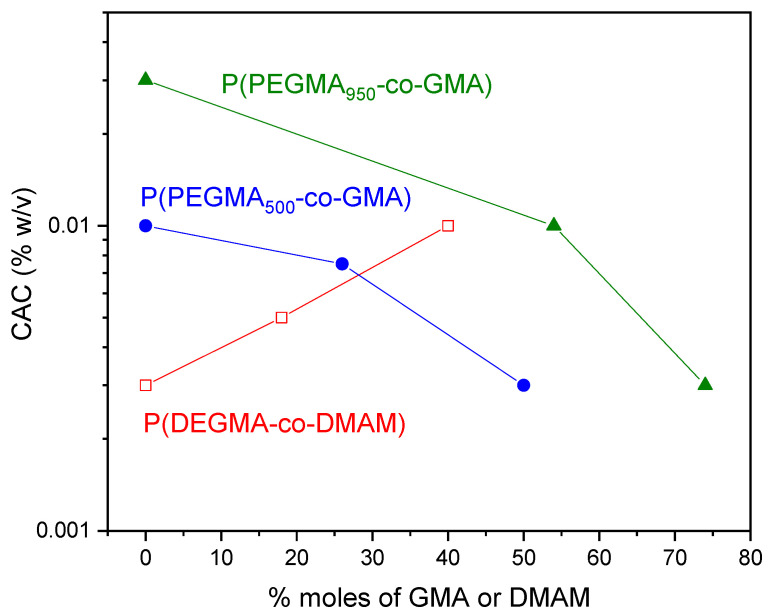
CAC vs. molar percentage of DMAM or GMA for P(DEGMA-co-DMAMy) (□), P(PEGMA_500_-co-GMAy) (●), and P(PEGMA_950_-co-GMAy) (▲).

The CAC values of the polymers, determined as the concentration of the sudden increase in the emission intensity are presented in Figure 5 versus the molar percentage of DMAM or GMA comonomers. All polymers exhibited CAC values comparable to reported CAC values for other OEGMA-based amphiphilic polymers [44]. It is worth noting that the homopolymers also present a self-association behavior, and CAC increases with the number of repeating ethylene oxide units in the OEGMA macromonomers. This can be explained by the change in the balance between hydrophilic and hydrophobic moieties in the molecular structure of POEGMA homopolymers. In fact, the apolar carbon–carbon backbone causes a hydrophobic effect while the ether oxygens of EG provide stabilization in water due to hydrogen bonding with water [24,45]. 

The introduction of the hydrophobic GMA comonomer in the copolymer chain leads to a decrease in CAC for the P(PEGMA_500_-co-GMAy) and P(PEGMA_950_-co-GMAy) copolymers, as compared to the CAC values of the PPEGMA_500_ and PPEGMA_950_ homopolymers, respectively. As seen more clearly in the case of P(PEGMA_500_-co-GMAy) copolymers, the CAC decrease is indeed remarkable, and it may be of about one order of magnitude for high GMA contents. In the case of the P(PEGMA_x_-co-GMAy) copolymers, the incorporation of GMA units results in a shift of the hydrophobic/hydrophilic balance towards hydrophobicity. This enhanced hydrophobicity, along with the hydrophobic effect due to the apolar carbon–carbon backbone of the PEGMA segments, probably leads to self-organization in nanostructures with hydrophobic GMA/apolar PEGMA backbones, stabilized in the aqueous media by the hydrophilic EG pendant chains. In contrast, the copolymerization of OEGMAs with the non-ionic and hydrophilic DMAM comonomer results in an increase in CAC. Indeed, the CAC of the homopolymer of PDEGMA (0.003% *w*/*v*) becomes three times higher when the DMAM content of the copolymer is 40%mol. It is thus clear that adjusting the hydrophobic or hydrophilic content of the copolymers is an efficient way to control CAC.

### 3.3. Thermo-Responsiveness in Aqueous Solution

**Figure 6:**
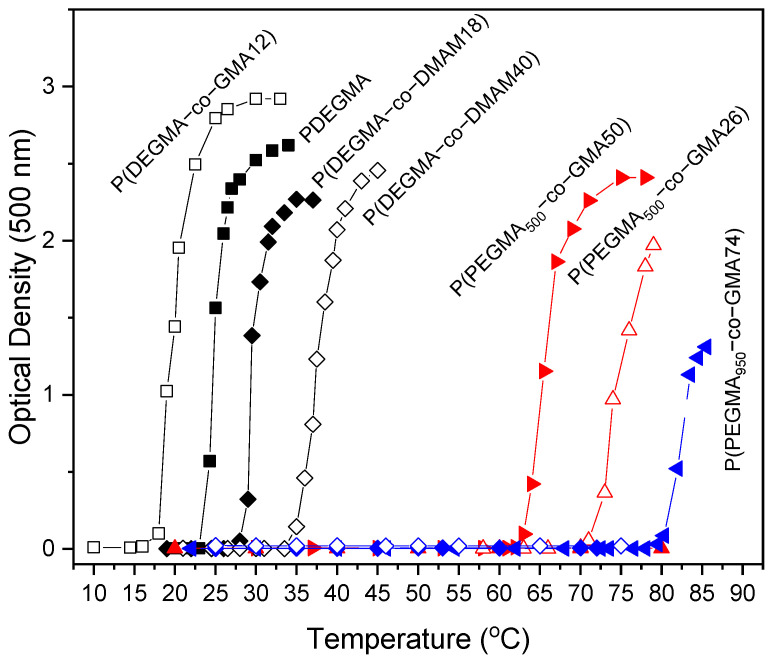
Optical density vs. temperature of PDEGMA (■), P(DEGMA-co-GMA12) (□), P(DEGMA-co-DMAM18) (♦), P(DEGMA-co-DMAM40) (◊), PPEGMA_500_ (▲), P(PEGMA_500_-co-GMA26) (∆), P(PEGMA_500_-co-GMA50) (►), PPEGMA_950_ (♦), P(PEGMA_950_-co-GMA54) (◊), and P(PEGMA_950_-co-GMA74) (◄).

Since POEGMAs are thermo-responsive polymers with lower critical solution temperature (LCST) behavior in water, herein, we studied the thermo-responsiveness of water-soluble OEGMA-based homopolymers and copolymers. In Figure 6, the variation in the optical density with temperature for aqueous solutions (0.2% *w*/*v*) of POEGMA homopolymers and OEGMA-based copolymers is presented. The aqueous solutions of PPEGMA_500_ and PPEGMA_950_ homopolymers and the P(PEGMA_950_-co-GMA54) copolymer are transparent throughout the entire temperature range applied in this work (heating was applied up to ~85 °C), indicating that they did not show thermo-sensitive macroscopic phase separation behavior for the concentration of 0.2% *w*/*v* studied. This finding is reported in the literature as well [26,46] and it can be attributed to the fact that the number of ethylene glycol groups in the side chains is sufficiently high to overcome the hydrophobicity of the backbone, thus leading to hydrophilic homopolymers, with them probably having a cloud point higher than the upper limit of the temperature range applied herein.

In contrast, all other systems studied turn sharply turbid upon heating at a temperature characteristic of each polymer, reflecting an LCST-type thermo-responsiveness. 

**Figure 7:**
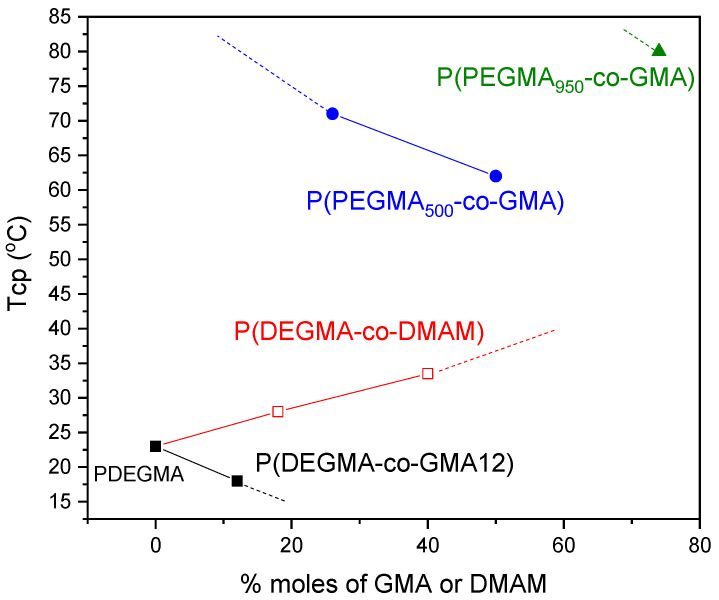
Tcp vs. molar percentage of DMAM or GMA for P(DEGMA-co-GMAy) (■), P(DEGMA-co-DMAMy) (□), P(PEGMA_500_-co-GMAy) (●), and P(PEGMA_950_-co-GMAy) (▲).

The cloud point temperature (Tcp) (defined as the temperature of the onset of turbidity, reflecting the transmittance decrease) is presented in Figure 7, as determined from the data shown in Figure 6. As seen, in contrast with the respective homopolymers, the solutions of P(PEGMA_950_-co-GMA74), P(PEGMA_500_-co-GMA26) and P(PEGMA_500_-co-GMA50) copolymers turn from transparent to cloudy upon heating above 80 °C (for P(PEGMA_950_-co-GMA74)), 71 °C (for P(PEGMA_500_-co-GMA26)), and 62 °C (for P(PEGMA_500_-co-GMA50)). The incorporation of hydrophobic GMA units into the P(PEGMA_x_-co-GMAy) chains influences the hydrophobic/hydrophilic balance, rendering the copolymer more hydrophobic and leading to the Tcp decrease. Moreover, the influence of the number of repeating ethylene glycol groups in OEGMA side chains is revealed when comparing copolymers with a similar GMA content, for example, P(PEGMA_500_-co-GMA50) and P(PEGMA_950_-co-GMA54). It can be seen that the 54% moles GMA content is not enough to render a thermo-responsive behavior for the second copolymer with the longer side chains. In fact, for PEGMA_950_-based copolymers, a higher content of hydrophobic GMA units (74% moles, P(PEGMA_950_-co-GMA74) copolymer) is necessary to display a cloud point at around 80 °C. On the other hand, the 50% moles GMA content in P(PEGMA_500_-co-GMA50) suffices to observe a lower cloud point (Tcp = 62 °C), in agreement with other findings in the literature. For example, for copolymers of PEGMA_500_ with dodecyl methacrylate or butyl methacrylate with 50% moles content in these hydrophobic units, cloud points at 61–62 °C have been found [47].

In contrast with PPEGMA_500_ and PPEGMA_950_ homopolymers, the PDEGMA homopolymer already exhibits thermo-sensitivity at low temperatures and the initially transparent aqueous PDEGMA solution turns turbid upon heating above 23 °C, close to the value of 26 °C reported in the literature [14,24]. As expected, the incorporation of rather low content of hydrophobic GMA units in the P(DEGMA-co-GMA12) copolymer leads to a Tcp decrease of about 5 °C. On the other hand, the incorporation of the hydrophilic DMAM units shifts the hydrophobic/hydrophilic balance toward hydrophilicity, resulting in enhanced water solubility and higher Tcp values. Thus, the cloud point of the P(DEGMA-co-DMAMy) solutions is gradually shifted to higher temperatures (28 °C and 33.5 °C for P(DEGMA-co-DMAM18) and P(DEGMA-co-DMAM40), respectively) as compared to the PDEGMA homopolymer. Similar trends have been found in the literature for copolymers of DEGMA with more hydrophilic comonomers, like 2-(dimethylamino)ethyl methacrylate (DMAEMA) [48].

It must be noted that the thermo-responsiveness of all samples was reversible, and the solutions returned from turbid to transparent upon cooling the systems under study. Moreover, the samples that turned turbid upon heating did not exhibit any polymer precipitation or macroscopic phase separation. This is due to the water solubility offered to the polymers by the OEGMA macromonomers (hydrogen-bonding interactions of the pendant poly(ethylene oxide) chains with the water molecules). Overall, these results indicate that the Tcp can be tuned to the desired temperature by incorporating different molar factions of either the hydrophobic GMA or the hydrophilic DMAM units in the copolymers’ chains. 

**Figure 8:**
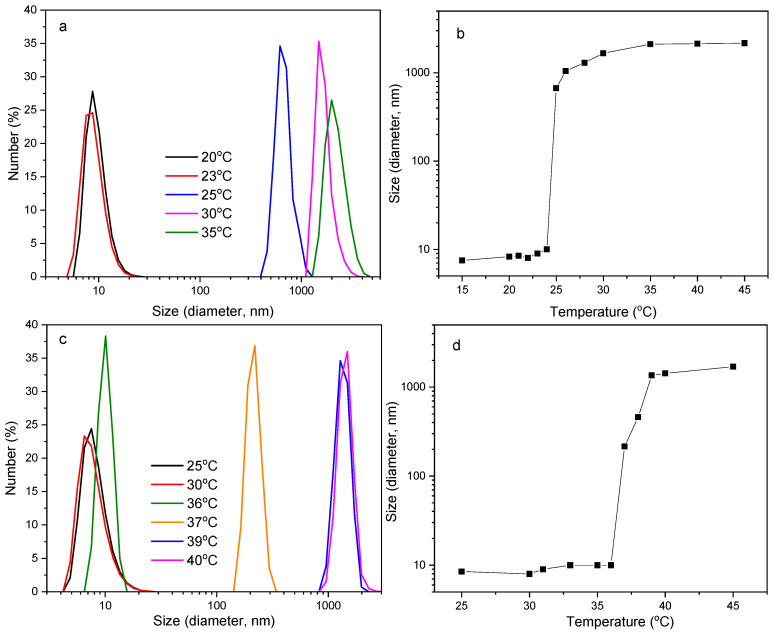
Temperature-dependent DLS size distribution and hydrodynamic diameter vs. temperature of aqueous (**a**,**b**) PDEGMA and (**c**,**d**) P(DEGMA-co-DMAM40) solutions at 0.2% *w*/*v* polymer concentrations.

The temperature-induced changes in the size of the thermo-responsive PDEGMA homopolymer and the statistical P(DEGMA-co-DMAM40) copolymer were also followed by DLS (Figure 8). In the case of the PDEGMA homopolymer (Figure 8a,b), it can be observed that below 23 °C, the homopolymer chains assembled probably in unimers with sizes ~10 nm in diameter. Above 23 °C, large aggregates with sizes > 400 nm (at 25 °C) are observed, while the solution becomes cloudy. Upon further heating, the homopolymer chains assemble into larger hydrophobic aggregates with a hydrodynamic size >1000 nm. Similar results were reported in Ramírez-Jiménez’s study on P(OEGMA-co-DEGMA) statistical polymers [32].

In the case of the P(DEGMA-co-DMAM40) statistical copolymer (Figure 8c,d), the copolymer chains form assemblies with small sizes varying from ~5–10 nm for temperatures up to 36 °C. At 37 °C and 38 °C, copolymer assemblies with higher size (~200 nm and 460 nm, respectively) are observed, accompanied by the formation of a turbid solution. Upon further heating above this temperature, the copolymer chains assembled into hydrophobic aggregates with a hydrodynamic size ~1500 nm. For both PDEGMA and P(DEGMA-co-DMAM40) polymers, it seems that the polymeric chains self-fold intramolecularly into unimers at low temperatures (below their Tcp) while at elevated temperatures, they form intermolecular multichain aggregates. 

The size of the polymer aggregates increases abruptly by increasing the temperature above the Tcp, probably due to two facts: (1) weakening of hydrogen bonds at higher temperatures, which inhibits polymer solubilization favoring further aggregation, and (2) the tendency of the polymer chain conformations to minimize their contact with the surrounding water molecules or to interact with the pre-existing copolymer aggregates, leading to transition toward micrometer-sized globular-like structures [49]. Additionally, it can be concluded that statistical incorporation of the hydrophilic DMAM units into the structure of the copolymer induces a shift of the Tcp to a higher value (37 °C) than that of the PDEGMA homopolymer (25 °C). The Tcp values determined via DLS are a bit higher than the data from the turbidity measurements shown in Figure 7 (23 °C for PDEGMA and 33.5 °C for P(DEGMA-co-DMAM40)).

### 3.4. Effect of Salts on the Thermo-Sensitive Behavior 

Both the cation and anion of a salt are considered to influence the solubility behavior of the macromolecules. The ordering of anions according to Hofmeister [50] is the following, with decreasing denaturation ability from left to right: CO_3_^2−^ >  SO_4_^2− ^ >   S_2_O_3_^2− ^ > HPO_4_^2−^ >  H_2_PO4^−^  >  F^−^  > Cl^−^ >  Br^−^ > NO_3_^−^  > I^−^  > ClO_4_^−^ > SCN^−^. Salts are categorized into two groups, kosmotropic and chaotropic ones. It is well-known that the presence of salts has a high impact on the thermo-responsive behavior of polymers in aqueous solution [51]. In the presence of kosmotropic salts, a salting out effect occurs, where fewer water molecules are available to hydrate the polymer, and therefore, the polymer’s solubility as well as LSCT decrease. On the other hand, in the presence of chaotropic salts, salting in effect is responsible for the increase in water molecules that can further hydrate the polymer, thus increasing the LSCT. For example, it has been shown that the cosmotropic SO_4_^2−^ salts of the Hoffmeister series decrease the LCST of random and gradient copolymers of di- and oligo-(ethylene oxide) methacrylate in water and therefore demonstrate a salting-out effect, while the chaotropic SCN^−^ salts increase the LCST and show a salting-in effect [33]. 

**Figure 9:**
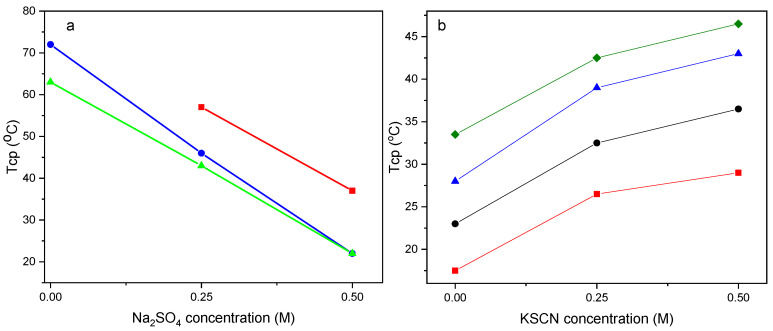
Tcp vs. salt concentration: (**a**) PPEGMA_500_ (squares), P(PEGMA_500_-co-GMA26) (circles), and P(PEGMA_500_-co-GMA50) (triangles), in the absence or presence of Na_2_SO_4_, and (**b**) PDEGMA (circles) and P(DEGMA-co-GMA12) (squares), P(DEGMA-co-DMAM18) (triangles), and P(DEGMA-co-DMAM40) (rhombi), in the absence or presence of KSCN.

Since the cloud point of PEGMA_500_-based (co)polymers is high, we chose a typical cosmotropic salt, namely Na_2_SO_4_. On the other hand, in the case of DEGMA-based (co)polymers, exhibiting a low Tcp, the chaotropic KSCN salt was chosen. The variation of the optical density with the temperature of both copolymer series in the presence of the respective salt is shown in Figure S2. From this figure, the Tcp values of the studied polymer solutions, presented in Figure 9, were determined. 

In Figure 9a, the effect of SO_4_^2−^ anions on the thermo-responsivity of the PEGMA_500_-based polymers is presented. In this case, sulfate anions hydrate and attract water molecules, leading to the dehydration of polymer chains and exposing the hydrophobic parts. Consequently, hydrophobic effects and polymer aggregation are promoted while the solution of PPEGMA_500_ in pure water (as already shown in Figure 6) did not display a Tcp; it can be seen that the presence of 0.25 M and 0.5 M of Na_2_SO_4_ provides thermo-responsiveness to the homopolymer. Moreover, the presence of Na_2_SO_4_ causes a decrease in the Tcp of P(PEGMA_500_-co-GMA26) and P(PEGMA_500_-co-GMA50) copolymer solutions. On the other hand, the presence of the chaotropic agent KSCN will lead to the weakening of hydrophobic interactions and thus enhance the solubility of hydrophobic domains. This is, indeed, shown in Figure 9b, where the influence of KSCN concentration on the Tcp of aqueous solutions of the PDEGMA homopolymer, as well as P(DEGMA-co-GMA12) and P(DEGMA-co-DMAMy) copolymers, is presented. As seen, a systematic gradual increase in the Tcp is observed as the KSCN concentration increases. This trend stands for all systems studied, shifting to higher temperatures with the decreasing hydrophobicity or increasing hydrophilicity of the (co)polymer. Thus, Tcp values about 10–15 °C higher are found when the solution contains 0.5 M KSCN, as compared to the Tcp values of the respective samples in pure water. 

### 3.5. Functional Character of P(PEGMA-co-GMA) Copolymers 

**Figure 10:**
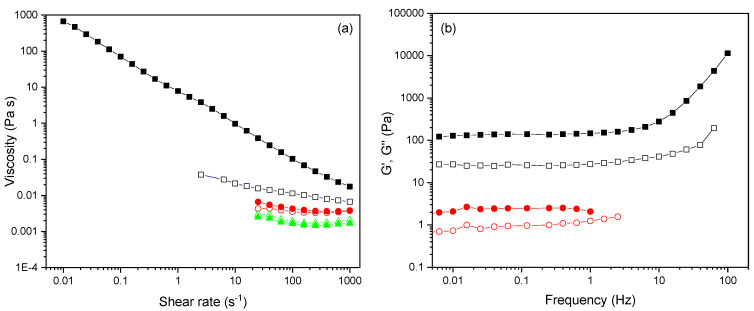
(**a**) Flow curves of viscosity versus shear rate for P(PEGMA_950_-co-GMA54) copolymer solution (circles), silica nanoparticles (triangles), and P(PEGMA_950_-co-GMA54)/silica nanoparticle suspensions (squares) at RT conditions (open symbols) and after heating overnight at 80 °C (closed symbols); (**b**) storage (G′, closed symbols) and loss (G″, open symbols) moduli versus frequency for P(PEGMA_950_-co-GMA54)/silica nanoparticle suspensions at RT conditions (circles) and after heating overnight at 80 °C (squares).

In addition to controlled amphiphilicity and thermo-sensitivity, the GMA-based copolymers designed in this work are also functional due to the reactivity of GMA toward nucleophiles, like hydroxyl, carboxylic, or amine groups. To demonstrate this additional functionality, we explore herein the possibility of our copolymers reacting with the surface OH groups of silica nanoparticles, as a proof of concept. In fact, in our research group, we have taken advantage of the reaction of GMA-based copolymers with carboxylic or amine groups for the development of functional membranes or coatings [52,53,54]. In these studies, we have observed that, unlike amines, the reaction of the carboxylic groups of acrylic acid with the epoxide group of GMA occurs only after heating at 80 °C, leading to cross-linked materials [52]. Similarly, according to the literature, for the chemical modification of silica nanoparticles with GMA, thermal treatment at high temperature is employed [55,56], apparently as a consequence of the weak nucleophilicity of hydroxyl groups. Having this in mind, we proceeded with the present rheology study after thermal treatment at 80 °C. P(PEGMA_950_-co-GMA54) was chosen for the initial tests, as it is the copolymer with the highest composition in GMA; consequently, it has the highest content in epoxide groups. At the same time, it is water soluble and exhibits the highest cloud point temperature of all systems studied herein. This way, we avoid possible implications from phase separation phenomena at the temperature applied for thermal treatment. Therefore, an aqueous mixture of P(PEGMA_950_-co-GMA54) with silica nanoparticles was evaluated through rheology studies (Figure 10). As seen in Figure 10a, the mixture has low viscosity after standing overnight at room temperature. On the contrary, when the aqueous mixture is heated overnight at 80 °C, it becomes highly viscous, displaying strong shear-thinning characteristics. This remarkable viscosity enhancement (about two orders of magnitude at 10 s^−1^) can be attributed to the reaction of GMA groups with the surface OH groups of silica nanoparticles at high temperature. Consequently, at the concentration studied, the nanoparticles act as cross-links, forming a hydrogel. Indeed, when the pure polymer P(PEGMA_950_-co-GMA54) and the bare silica nanoparticles were tested under the same conditions, no changes were observed before and after heating overnight at 80 °C, verifying that the presence of GMA groups is necessary to form the nanoparticle-mediated cross-linked hydrogel. The frequency dependence of viscoelastic moduli G′ and G″ of the P(PEGMA_950_-co-GMA54)/silica composite is plotted in Figure 10b. While the storage modulus G′ and the loss modulus G″ exhibit low values when the mixture has not been heated, after heating the P(PEGMA_950_-co-GMA54)/silica mixture at 80 °C overnight, the G′ and G″ moduli are nearly constant (within the frequency region 0.01–10 Hz), with G′ > G″ underlying the solid-like (gel-like) behavior. 

## 4. Conclusions

In the present work, statistical OEGMA-based homopolymers and copolymers were successfully synthesized through free radical polymerization. We mostly focused on copolymers with the functional hydrophobic GMA monomer, while copolymers with the nonionic hydrophilic DMAM monomer were also synthesized for the sake of comparison. All polymers were characterized and investigated in terms of their self-organization and thermo-responsive properties. The CAC of all polymers is controlled by their hydrophilic/hydrophobic balance. Thus, the P(PEGMA_500_-co-GMAy) and P(PEGMA_950_-co-GMAy) copolymers exhibit lower CAC values than the PPEGMA_500_ and PPEGMA_950_ homopolymers due to the incorporation of hydrophobic GMA units. On the other hand, in the case of P(DEGMA-co-DMAMy) copolymers, it has been shown that the CAC of the copolymers increases with increasing DMAM content. Similarly, the Tcp of the polymers is effectively controlled by the content of the copolymers in DMAM or GMA units. Indeed, the Tcp values of P(OEGMA-co-GMAy) copolymers shift to lower temperatures, whereas those of P(DEGMA-co-DMAMy) copolymers shift to higher temperatures, as compared to the respective OEGMA homopolymers. Moreover, it has been shown that the thermo-sensitivity of all polymers depends on the ionic strength of the aqueous solution. In fact, the presence of the kosmotropic SO_4_^2−^ anions in the aqueous polymer solutions leads to a decrease in the Tcp values, while increased Tcp values are found in the presence of the chaotropic SCN^−^ anions. Finally, the GMA-containing copolymers are also functional, as has been shown through the formation of a hydrogel after heating aqueous mixtures of these copolymers with silica nanoparticles. 

Overall, functional polymeric materials with regulated and on-demand thermo-responsive properties were effectively designed in the present work. Such polymers are promising for advanced applications in several fields, like drug delivery or other technological applications related to controlled rheology, “smart” membranes, or coatings.

## Figures and Tables

**Table 1:** Characterization results of the studied polymers.

Polymer	Feed Composition, %mol GMA or DMAM	Composition, %mol GMA or DMAM from ^1^H-NMR
PDEGMA	0	0
P(DEGMA-co-GMA12)	13	12
P(DEGMA-co-DMAM18)	17	18
P(DEGMA-co-DMAM40)	31	40
PPEGMA_500_	0	0
P(PEGMA_500_-co-GMA26)	28	26
P(PEGMA_500_-co-GMA50)	50	50
PPEGMA_950_	0	0
P(PEGMA_950_-co-GMA54)	50	54
P(PEGMA_950_-co-GMA74)	70	74

## Data Availability

Data is contained within the article or Supplementary Material.

## Supplementary Materials

The following supporting information can be downloaded at: https://www.mdpi.com/article/10.3390/polym16111456/s1. Figure S1: ^1^H-NMR spectra of the P(PEGMA_500_-co-GMAy) copolymers along with the respective spectra of the PPEGMA_500_ and PGMA homopolymers; Figure S2: Optical density vs. temperature of polymers in the absence or presence of Na_2_SO_4_ or KSCN.
