# Supplementary material for: Controlled Amphiphilicity and Thermo-Responsiveness of Functional Copolymers Based on Oligo(Ethylene Glycol) Methyl Ether Methacrylates
Source: Polymers (Basel). 2024 May 22;16(11):1456. doi: 10.3390/polym16111456 (PMC11174388; doi:10.3390/polym16111456)
Supplement: Supplementary file 1 [file polymers-16-01456-s001.zip › polymers-2995834-supplementary.pdf]

Supplementary Material

Controlled Amphiphilicity and Thermo–responsiveness of Functional Copolymers Based on Oligo(Ethylene Glycol) Methyl Ether Methacrylates

Aggeliki Christopoulou[1], Charalampos Kazamiakis[1], Zacharoula Iatridi[2,*], Georgios Bokias[1,3]

[1] Department of Chemistry, University of Patras, GR-26504 Patras, Greece; up1064175@ac.upatras.gr (A.C); up1073632@ac.upatras.gr (C.K); bokias@upatras.gr (G.B)
[2] Department of Materials Science, University of Patras, GR-26504 Patras, Greece; iatridi@upatras.gr (Z.I)
[3] Foundation for Research and Technology Hellas (FORTH), Institute of Chemical Engineering and High Temperature Chemical Processes, 26504 Patras, Greece
* Correspondence: iatridi@upatras.gr (Z.I)

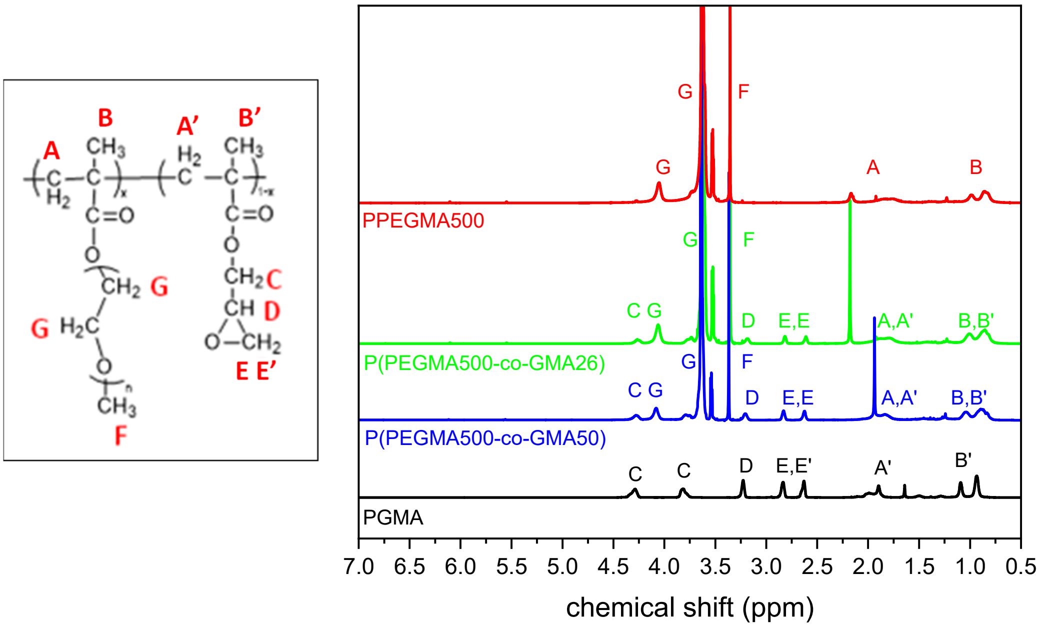

Figure S1. ^{1}H–NMR spectra of the P(PEGMA$_{500}$–co–GMAy) copolymers along with the respective spectra of the PPEGMA500 and PGMA homopolymers.

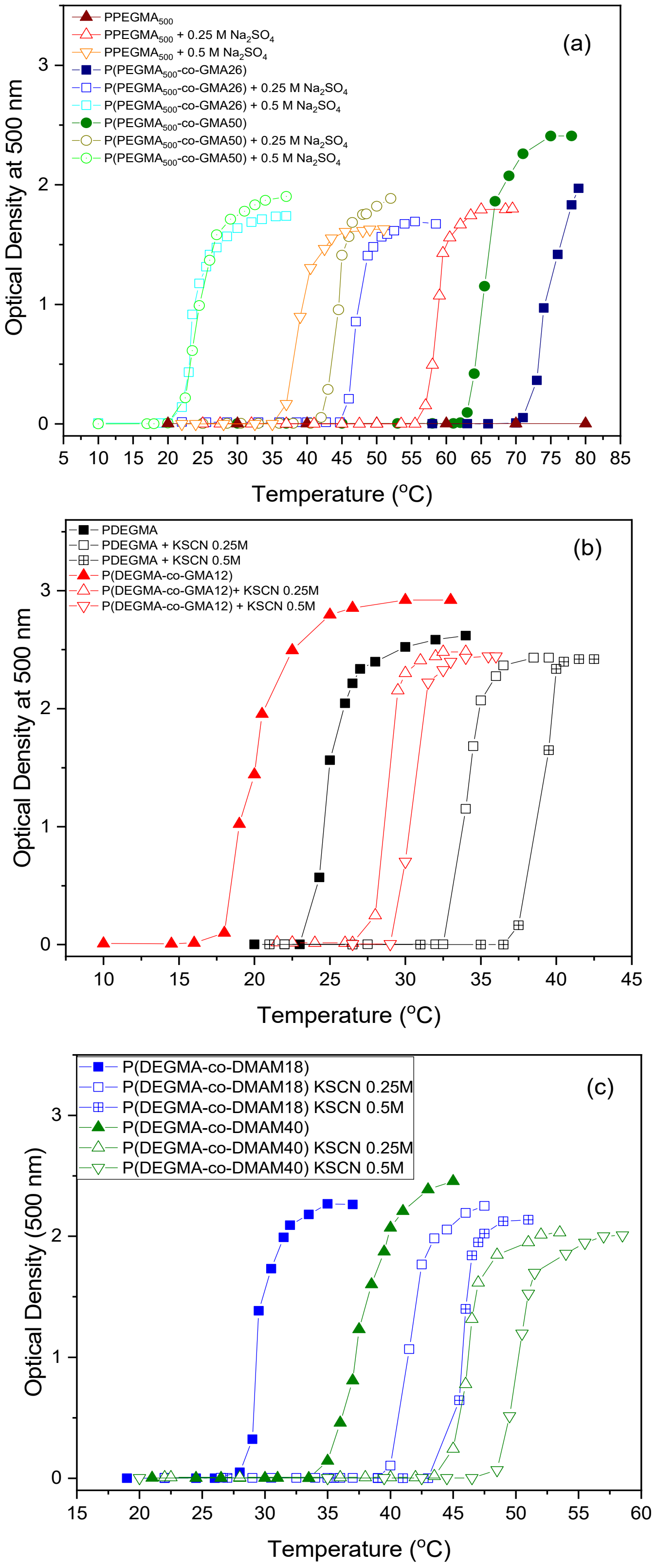

Figure S2. Optical density vs temperature of a) $PPEGMA_{500}$ (triangle symbols), P($PEGMA_{500}$–co–GMA26) (square symbols), and P($PEGMA_{500}$–co–GMA50) (circle symbols), in the absence or presence of Na_2SO_4; b) PDEGMA (black

square symbols) and P(DEGMA−co−GMA12) (red triangle symbols), in the absence or presence of KSCN; c) P(DEGMA−co−DMAM18) (black square symbols) and P(DEGMA−co−DMAM40) (blue triangle symbols), in the absence or presence of KSCN.
